# The Role of Reactive Oxygen Species in *Anopheles aquasalis* Response to *Plasmodium vivax* Infection

**DOI:** 10.1371/journal.pone.0057014

**Published:** 2013-02-18

**Authors:** Ana C. Bahia, José Henrique M. Oliveira, Marina S. Kubota, Helena R. C. Araújo, José B. P. Lima, Claudia Maria Ríos-Velásquez, Marcus Vinícius G. Lacerda, Pedro L. Oliveira, Yara M. Traub-Csekö, Paulo F. P. Pimenta

**Affiliations:** 1 Laboratório de Biologia Molecular de Parasitas e Vetores, Instituto Oswaldo Cruz, Fiocruz, Rio de Janeiro, Brazil; 2 Laboratório de Bioquímica de Artrópodes Hematófagos, Instituto de Bioquímica Médica, Programa de Biologia Molecular e Biotecnologia, Universidade Federal do Rio de Janeiro, Rio de Janeiro, Brazil; 3 Laboratório de Entomologia Médica, Instituto René Rachou, Belo Horizonte, Brazil; 4 Laboratório de Fisiologia e Controle de Artrópodes Vetores, Instituto Oswaldo Cruz, Fiocruz, Rio de Janeiro, Brazil; 5 Laboratório de Biodiversidade em Saúde, Centro de Pesquisa Leônidas & Maria Deane, Fiocruz, Manaus, Brazil; 6 Fundação de Medicina Tropical Dr. Heitor Vieira Dourado, Manaus, Brazil; 7 Instituto Nacional de Ciência e Tecnologia em Entomologia Molecular, Rio de Janeiro, Brazil; Centro de Pesquisas René Rachou, Brazil

## Abstract

Malaria affects millions of people worldwide and hundreds of thousands of people each year in Brazil. The mosquito *Anopheles aquasalis* is an important vector of *Plasmodium vivax*, the main human malaria parasite in the Americas. Reactive oxygen species (ROS) have been shown to have a role in insect innate immune responses as a potent pathogen-killing agent. We investigated the mechanisms of free radicals modulation after *A. aquasalis* infection with *P. vivax*. ROS metabolism was evaluated in the vector by studying expression and activity of three key detoxification enzymes, one catalase and two superoxide dismutases (SOD3A and SOD3B). Also, the involvement of free radicals in the mosquito immunity was measured by silencing the catalase gene followed by infection of *A. aquasalis* with *P. vivax*. Catalase, SOD3A and SOD3B expression in whole *A. aquasalis* were at the same levels of controls at 24 h and upregulated 36 h after ingestion of blood containing *P. vivax*. However, in the insect isolated midgut, the mRNA for these enzymes was not regulated by *P. vivax* infection, while catalase activity was reduced 24 h after the infectious meal. RNAi-mediated silencing of catalase reduced enzyme activity in the midgut, resulted in increased *P. vivax* infection and prevalence, and decreased bacterial load in the mosquito midgut. Our findings suggest that the interactions between *A. aquasalis* and *P. vivax* do not follow the model of ROS-induced parasite killing. It appears that *P. vivax* manipulates the mosquito detoxification system in order to allow its own development. This can be an indirect effect of fewer competitive bacteria present in the mosquito midgut caused by the increase of ROS after catalase silencing. These findings provide novel information on unique aspects of the main malaria parasite in the Americas interaction with one of its natural vectors.

## Background

Malaria is an important health problem that affects millions of people and causes millions of deaths worldwide each year. In Brazil, this disease affects mainly the Northern region with approximately 450,000 cases per year, 85% of them being due to *Plasmodium vivax* [1]. We are investigating the interaction of *P. vivax* with *Anopheles aquasalis*, an important malaria vector in the coast of Brazil [2].

For malaria transmission to occur the parasite needs to complete a complex life cycle inside the insect vector that includes: differentiation of gametes, fertilization, passage through the midgut epithelial cells, establishment in the midgut basal lamina as oocysts, multiple cell divisions with breakdown of the oocysts and the release of hundreds of sporozoites into the hemolymph, invasion of the salivary gland, differentiation, and finally inoculation into a new vertebrate host [3]. During these steps the parasite interacts with diverse insect tissues causing activation of the mosquito powerful innate immune defenses, which are responsible for major parasite losses [4], [5].

The reactive oxygen species (ROS) are one class of effector implicated in insect innate immunity. ROS are multifunctional molecules involved in host defense, mitogenesis, hormone biosynthesis, apoptosis, necrosis, and gene expression [6], [7]. The importance of ROS in immune response was first described in phagocytic cells through ROS production by NADPH oxidases (NOX) followed by pathogen killing [8], [9]. To date, six human homologues of the NOX protein family (Nox-1, Nox-3, Nox-4, Nox-5, Duox-1 and Duox-2) have been identified in various non-phagocytic cells [7]. Homologues of some of these proteins were also discovered in nematodes, fruit flies, green plants, fungi and slime molds [10]. The Dual Oxidases (DUOXs) are important in hormone production, extracellular matrix production and host defense [11]. ROS producing DUOX proteins were described in *Drosophila melanogaster* and *Anopheles gambiae* after pathogen challenges [9], [12]–[15]. In *A. gambiae*, DUOX proteins, together with a peroxidase, are responsible for preventing a strong immune activation by producing a dityrosine network, which decreases gut permeability to immune elicitors [16]. This mucous protection may prevent the deleterious effect of the immune response to the host itself and to commensal gut bacteria.

Luckhart and collaborators [17], [18] described an increase of the free radical nitric oxide as well as of nitric oxide synthase (NOS) in *Anopheles stephensi* after *Plasmodium berghei* invasion of epithelial cells. Also, *A. gambiae* under high oxidative stress was more resistant to *Plasmodium* parasites and bacteria [19], [20]. This resistance profile was reverted when these insects were subjected to an antioxidant diet. Furthermore, after blood ingestion and even more after *Plasmodium* infection, the expression of some detoxification enzymes increased significantly.

In spite of ROS being beneficial for parasite clearance, they are potentially toxic to the host itself. For this reason, the lifespan of these molecules must undergo a fine tuned regulation, which is accomplished through the action of antioxidant enzymes, such as superoxide dismutase (SOD) and catalase, as well as the control of ROS generation. SODs transform superoxide (O_2_^−•^) into hydrogen peroxide (H_2_O_2_) and catalase detoxifies H_2_O_2_ into water and oxygen. Other molecules as vitamin C and uric acid are also antioxidant components utilized by the organisms to neutralize deleterious effects of high levels of ROS.

Following evidence for a role of ROS in *A. stephensi* and *A. gambiae* immunity, we investigated the recruitment of ROS as an immune defense of the Brazilian natural malaria vector *A. aquasalis* against *P. vivax*, the main human malaria parasite in the Americas. It is noteworthy that the *P. vivax* utilized in our experiments is from human infected patients, bringing our results closer to a natural situation. We also investigated the mechanisms used to minimize the harmful effects of the ROS generation by this mosquito. Our results revealed detoxification enzymes expression modulation and a possible manipulation of catalase enzyme by the *P. vivax* parasite in order to increase its development and survival.

## Materials and Methods

### Ethics statement

For the acquisition of *P. vivax* infected human blood, patients were selected among the people visiting the Health Center (Posto Estadual de Saúde da Vigilância em Saúde do Município de Iranduba, Distrito de Cacau Pirêra, Amazonas, Brazil) looking for malaria diagnosis and treatment during outbreaks. Diagnosis was performed by Giemsa stained blood smear. After positive diagnosis and visualization of gametocytes, patients were interviewed and inquired about the possibility of volunteer donation of a small amount of blood for research purposes. After verbal agreement, a term of consent was first read to the potential volunteers, with detailed verbal explanation, and, after final consent, signed by the patient. After this, one 200 µl sample of venous blood was drawn from each patient and placed in heparinized tubes. Blood samples were kept under refrigeration in an ice box (at approximately 15°C) for about 15 minutes, taken to the laboratory and used to feed *A. aquasalis*. Patient selection criteria were: to be *P. vivax* positive, to have about 4–8% of circulating gametocytes, determined by the National Institutes of Health international protocols, and to consent to be part of the research (consent form was approved by the Brazilian Ministry of Health, National Council of Health, National Committee of Ethics in Research (CONEP), written approval number 3726).

### Mosquito infection

*A. aquasalis* reared under controlled temperature and humidity [21] were blood-fed and infected by artificial feeding device. All insect infections were conducted in Manaus (Amazonas state) as described [22]. To prevent exflagellation of *P. vivax* microgametocytes, infective feeding was performed at 37°C. Mosquitoes were then transferred to a new cage and fed with 20% sucrose *ad libitum* until the experimental procedures. Infection was evaluated by PCR using a specific *Plasmodium* 18s rRNA gene as described [23].

### PCR using degenerate primers

Degenerate primers were designed based on conserved regions of SODs and catalase of *A. gambiae*, *A. stephensi*, *Aedes aegypti* and *D. melanogaster*, as previously described [24]. The cycles used in the PCR reaction were: two cycles of 1 min steps at 95, 55 and 72°C, and 95, 42 and 72°C followed by 30 cycles at moderate stringency (1 min steps at 95, 52 and 72°C) and a final 7 min extension at 72°C. Amplicons generated were cloned using pGEM®-T Easy Vector (Promega) and plasmids containing inserts were sequenced. All sequencing was performed using an ABI 3700 sequencer (Applied Biosystems) in the PDTIS/FIOCRUZ Sequencing Facility, Rio de Janeiro, Brazil.

### RACE and sequence analysis

SOD3A, SOD3B and Catalase 5′ and 3′ cDNA ends were obtained using the Smart cDNA RACE amplification kit (Becton Dickinson Clontech). SODs and catalase full cDNAs were obtained after assembling the sequences using the CAP3 program [25] and aligning these with other insect sequences. Neighbor-joining phylogenetic reconstructions based on Kimura 2-parameter (K2-p) distance matrices, with 1000 bootstrap replications, using the MEGA 4.0 software [26] were performed with the sequences of *A. aquasalis* and other insects.

### Quantitative PCR (qPCR)

qPCR was performed with cDNA from whole bodies or midguts from *A. aquasalis* submitted to different experimental conditions (sugar-fed males and females, blood-fed and *P. vivax* infected females). Previous to the cDNA synthesis, RNAs extracted with TRIzol® Reagent (Invitrogen) were treated with RQ1 DNAse free-RNAse (Promega). Syber Green fluorescent probe (Applied Biosystems) was used to reveal the amplification rate of catalase and SODs. The qPCR reactions were performed in an ABI 7000 real time PCR system (Applied Biosystems). The PCR cycles used were 50°C 2 min, 95°C 10 min, 95°C 15 sec and 63°C 1 min for 35 times for all reactions. The primer sequences were: SOD3AFwd 5′ GTGGAGAGGCAACCCCTTGAGAA 3′ and SOD3ARev 5′ GGTCGATCTTAGCGTGAAGCAGATT 3′, SOD3BFwd 5′ GTGGAGAGGCAACCCCTTGAGAA 3′ and SOD3BRev 5′ CTGATTCCAGGGTACATCGGTG 3′, and CatalaseFwd 5′ CGGACATGTTCTGGGACTTTATCT 3′ and CatalaseRev 5′ TTGCCCTCGGCGTTCACCAGCTTAA 3′, 16sFwd 5′ GGACTACCAGGGTATCTAATCCTGTT and 16sRev 5′ TCCTACGGGAGGCAGCAG T. The relative expression of the selected genes was based on gene expression CT difference formula [27]. Quantifications were normalized in relation to the housekeeping gene rp49 [28] and sugar-fed females were used as reference sample to calculate the relative expression. All experiments were performed using four to six biological replicates. The statistics method used in the analyses was ANOVA test with multiple comparisons of Tukey or Games-Howell. When the parametric model (ANOVA) was not adequate, we utilized the Kruskal-Wallis test. For the male versus female analyses the t-student or the Wilcoxon tests were utilized. All tests were performed with reliable level of 95% (α = 0.05). The statistical analyses were accomplished using the Graph pad Prism5®, R, software.

### Antioxidant enzymes activity

Three to six samples containing ten midguts of female *A. aquasalis* submitted to sugar-feeding, blood-feeding and infected blood-feeding were stored at −70°C in a cocktail of protease inhibitors (1 mM of Benzamidine, 1 mM of PMSF and 50 µg/µL of SBTI) until assayed. Guts of blood-fed insects were dissected in 50% ethanol for blood bolus removal. Catalase activity was determined by monitoring hydrogen peroxide consumption at 240 nm at room temperature according to Aebi [29]. SOD activity was measured based on the rate of cytochrome *c* reduction by O_2_^−**·**^ monitored at 550 nm and 25°C using the xanthine-xanthine-oxidase system as the source of O_2_^−^. [30]. Data were reported as the mean ± SEM. The statistics method used in the analysis was ANOVA test with Dunnett's Multiple Comparison Test or unpaired t-test. All tests were performed with reliable level of 95% (α = 0.05). The statistical analyses were accomplished using the Graph pad Prism5®, R, software.

### Catalase silencing and inhibition

The T7 Megascript kit (Ambion) was used to construct double stranded RNAs (dsRNAs) for *Catalase* (dsCat) and *ß-gal* (dsß-gal) from PCR-amplified fragments. Amplicons for dsß-gal were produced using plasmid templates and for dsCatalase by RT-PCR products, from sugar-fed female cDNA, giving rise to 544 bp and 466 bp fragments, respectively. Two rounds of PCR were necessary to amplify *ß-gal* and *Catalase*. The first PCR round was performed with primers containing a short adaptor sequence at the 5′ end (tggcgcccctagatg): ß-galFwd 5′ tggcgcccctagatgTGATGGCACCCTGATTGA 3′ and ß-galRev 5′ tggcgcccctagatgTCATTGCCCAGAGACCAGA 3′, dsCatalaseFwd 5′ tggcgcccctagatgCGTACAATCCGTTCGATCT 3′ and dsCatalaseRev 5′ tggcgcccctagatgACTGTTGCCTGCGAGAAGTT 3′. The PCR cycles used were 95°C for 3 min, 35 cycles of 95°C for 30 sec, 57°C for 45 sec and 72°C for 45 sec followed by 72°C for 7 min. For the second PCR reaction, two microliters of the first PCR product were used. The second round of PCR was utilized to insert the bacteriophage T7 DNA-dependent RNA polymerase promoters into the dsDNA templates. The cycle of the second round of PCR was the same utilized in the first reaction. The second round PCR primer used, which had the T7 and the adaptor sequences, was 5′ ccgTAATACGACTCACTATAGGtggcgcccctagatg 3′.

Sixty nine nanoliters of dsRNA for ß-gal and catalase diluted in water to a concentration of 3 µg/µL were introduced into the thorax of cold anesthetized 2–4 day old female mosquitoes by a nano-injector (Nanoject, Drummond) with glass capillary needles. The insects were maintained in an air incubator at 28°C and fed on sugar solution after the dsRNA injections. *P. vivax* infected blood was offered to the inoculated insects two to three days after the dsRNA injections. For catalase inhibition, 50 µL of 75 mM Aminotriazole or Phosphate buffer were added to 200 µL of *P. vivax* infected blood.

Oocyst counting was performed three to five days after infection. At least 50 guts of each experimental condition were dissected, stained with 2% Mercurechrome and observed under light microscopy. Three replicates of each experiment were performed. Oocyst numbers in dsCat-injected insects were compared to dsß-gal injected controls. The significance of gene silencing effect on oocyst loads between the experimental and control groups was determined by the Mann-Whitney statistical test.

### Hydrogen Peroxide measurements

H_2_O_2_ was measured using the Amplex Red® method as described elsewhere with minor modifications [31]. Briefly, the midgut epithelia of sugar-fed mosquitoes was dissected in PBS + BSA (2.5%) and kept in ice-cold PBS during sample collection. This step was followed by a 30 min incubation in PBS + Amplex Red (40 µM) + Horseradish Peroxidase (4 units) at room temperature and dim light with pools of 5 organs per tube. The experiments were performed three times with three biological replicates each. After the incubation period samples were spun down and fluorescence of the supernatant was immediately assessed (Ex/Em – 530/590 nm). Unspecific signal due to Amplex Red oxidation by the midgut epithelia (pools of 5 organs) in the absence of HRP was subtracted. The statistics method used in the analysis was unpaired t-test. All tests were performed with reliable level of 95% (α = 0.05). The statistical analyses were accomplished using the Graph pad Prism5®, R, software.

## Results

### Identification and characterization of antioxidant enzymes in *A. aquasalis*

**Figure 1:**
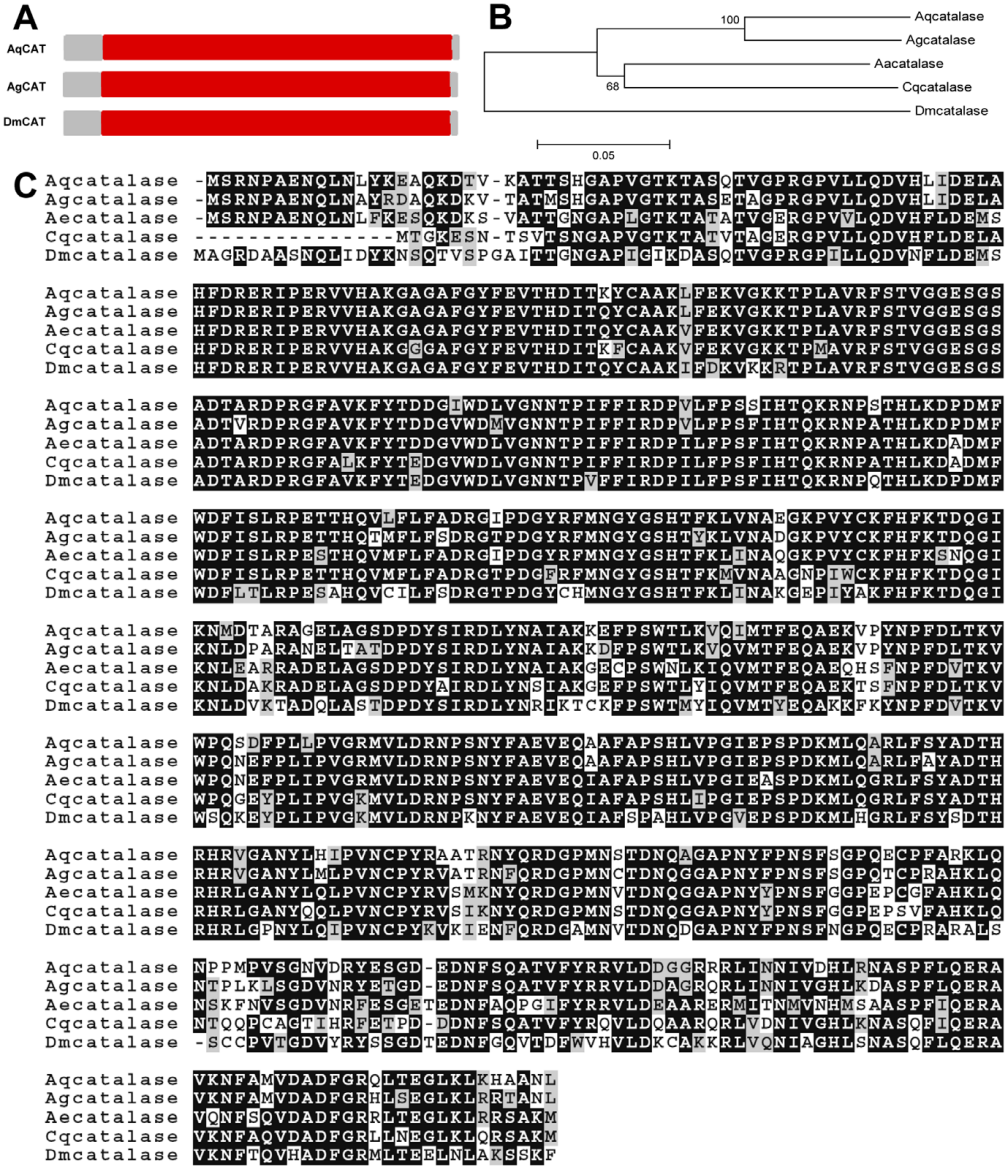
Characterization of Catalase cDNA. A: Schematic representation of *A. aquasalis* catalase (AqCAT) deduced protein. Red - clade 3 of the heme-binding catalase domain. B: Phylogenetic tree for catalase constructed based on the neighbor-joining method. C: Multiple aminoacid sequence alignment of insect catalase related proteins. Accession numbers of catalase sequences from: *A. aquasalis* (Aq) (HQ659100), *A. gambiae* (Ag) (XP_314995.4), *A. aegypti* (Aa) (XP_001663600.1), *Culex quinquefasciatus* (Cq) (XP_001848573.1) and *D. melanogaster* (Dm) (NP_536731.1).

cDNAs for two SODs and one catalase were amplified by PCR using degenerate primers. Expected fragments of 803 bp for catalase, 541 bp for SOD3A and 268 bp for SOD3B were obtained (data not shown). Smart Race PCR technique was utilized to amplify the full-length cDNAs. A 1989 bp full-length *A. aquasalis* catalase cDNA (AqCAT) was obtained, including a 1515 bp coding region, which translates into a 505 amino acid protein, as well as a 161 bp 5′ untranslated region (UTR) and 313 bp 3′ UTR (Figure S1). AqCAT is very similar to other insect catalases (Figure 1) giving rise to one long catalase domain (comprising the heme binding pocket and the NADPH binding site) also present in *A. gambiae* and *D. melanogaster* enzymes (Figure 1A). In addition, AqCAT bears 94% and 72% identity respectively with *A. gambiae* (XP_314995.4) and *D. melanogaster* (NP_536731.1) catalases (Figure 1B and 1C) and is not related to the immune-regulated catalase described in *D. melanogaster* (data not shown) [32].

**Figure 2:**
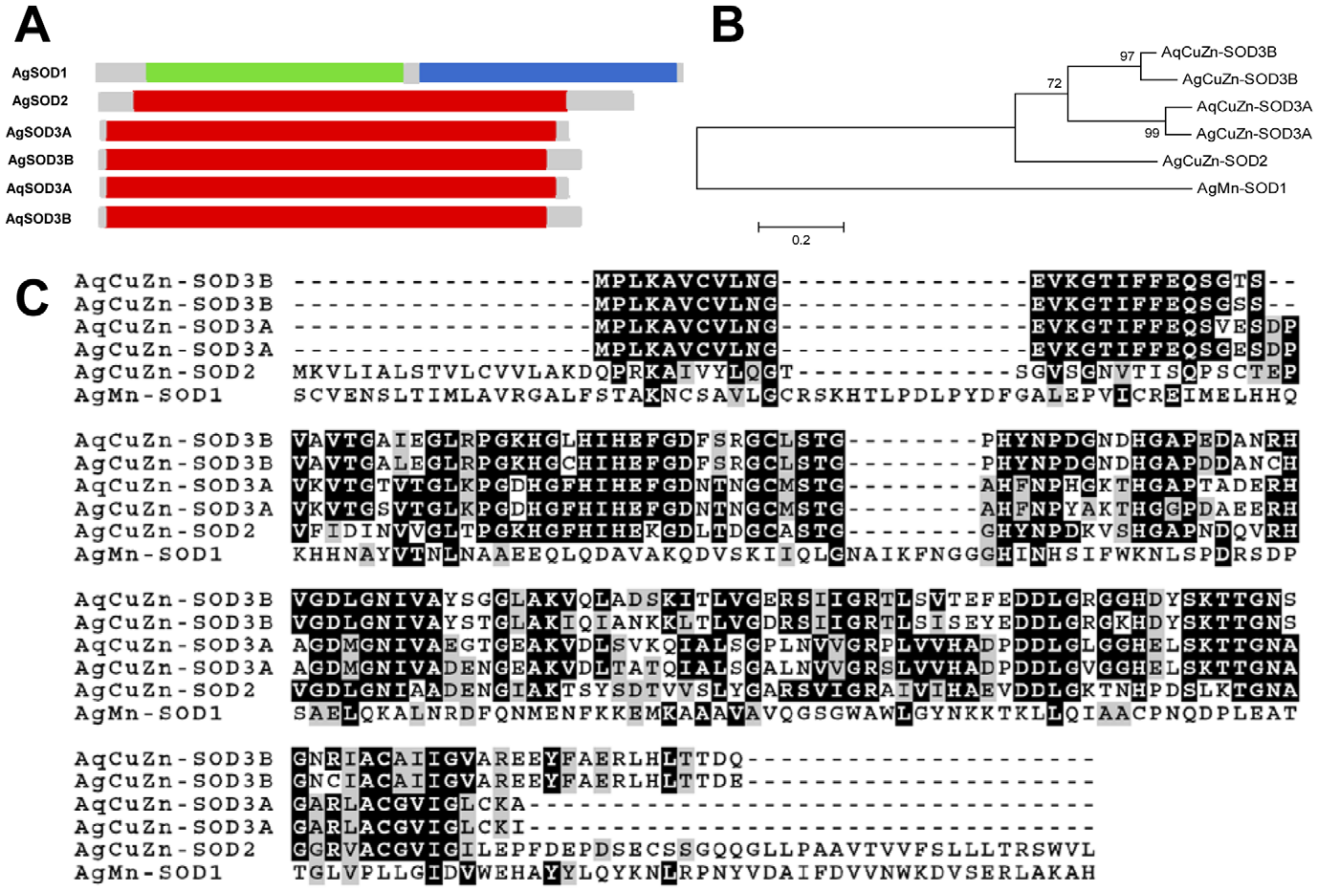
Characterization of SOD3A and SOD3B cDNA. A: Schematic representation of SOD3A (A) and 3B (B) protein from *A. aquasalis* (AqSOD3A and SOD3B). Green: iron/manganese superoxide dismutases alpha-hairpin domain; blue: iron/manganese superoxide dismutases C-terminal domain; red: Cu-Zn_superoxide_dismutase domain. B: Phylogenetic tree for SOD constructed based on the neighbor-joining method. C: Multiple aminoacid sequence alignment of mosquito SOD related proteins. Accession numbers of SOD sequences from: *A. aquasalis* (Aq) (SOD3A - HQ659101 and SOD3B HQ659102), *A. gambiae* (Ag) (SOD1 - XP_314490.3, SOD2 - XP_314137.4, SOD3A - XP_311594.2 and SOD3B - XP_001230820.1).

The full-length *A. aquasalis* SOD3A (AqSOD3A) cDNA sequence consists of 646 bp, including a 462 bp coding region, which encodes a 154 amino acid protein, as well as a 74 bp 5′ and 110 bp 3′ UTR (Figure S2A). The full-length *A. aquasalis* SOD3B cDNA (AqSOD3B) is 637 bp long including a 495 bp open reading frame (ORF), encoding a 165 amino acids protein, plus 63 bp upstream and 79 bp downstream UTRs (Figure S2B). The deduced AqSOD3A and AqSOD3B proteins have conserved Cu^2+^ and Zn^2+^ binding domains typically found in CuZn-superoxide dismutases (Figures S2A, S2B and 2A), bearing 94% and 97% identity with putative SOD3A (XP_311594.2) and SOD3B (XP_001230820.1) orthologous genes from *A. gambiae* (Figure 2B and 2C).

### Characterization of Catalase and SOD transcription and activity in *A. aquasalis*

**Figure 3:**
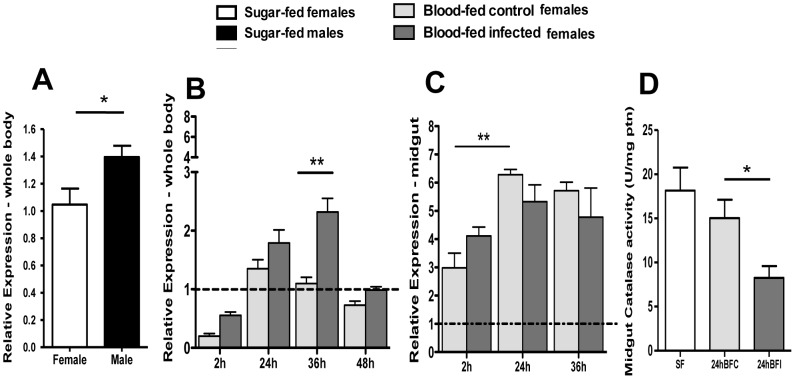
Expression levels of catalase in *A. aquasalis* according to gender and feeding regimens. A: mRNA relative expression of catalase in whole body of sugar-fed males and females; B and C: mRNA relative expression of catalase in the mosquito whole body and midgut, respectively, of sugar-fed females (dotted line), females after blood-feeding and after *P. vivax* infection; D: midgut catalase activity in sugar-fed (SF), 24 h after blood-feeding (BFC) and after *P. vivax* infection (BFI) reported as units per minute per micrograms of protein (U/mg ptn). Data are reported as the mean ± SEM. h – hours. * p<0.05, ** 0.03>p>0.01, *** p<0.01. In figure A the data was analyzed by t-student or the Wilcoxon tests, in figure B and C by ANOVA test with multiple comparisons of Tukey or Games-Howell or Kruskal-Wallis test with multiple comparisons of Dunn's, and in figure C by ANOVA test with Dunnett's Multiple Comparison Test.

Gene expression analyzes using cDNAs from whole *A. aquasalis* sugar fed males and females demonstrated that catalase transcription levels were higher in male mosquitoes (Figure 3A). To explore the putative involvement of this enzyme in malaria infection we performed a time course analysis of detoxification enzymes expression and activity in mosquitoes fed with *P. vivax*-infected or control blood. Catalase transcription was significantly upregulated in whole bodies of infected mosquitoes only at the 36 h point after feeding (Figure 3B). In the mosquito midgut, this enzyme was upregulated with the ingestion of blood, but was not modulated by infection with *P. vivax* (Figure 3C). Enzyme activity in the midgut was significantly reduced at 24 h in *P. vivax* infected mosquitoes (Figure 3D).

**Figure 4:**
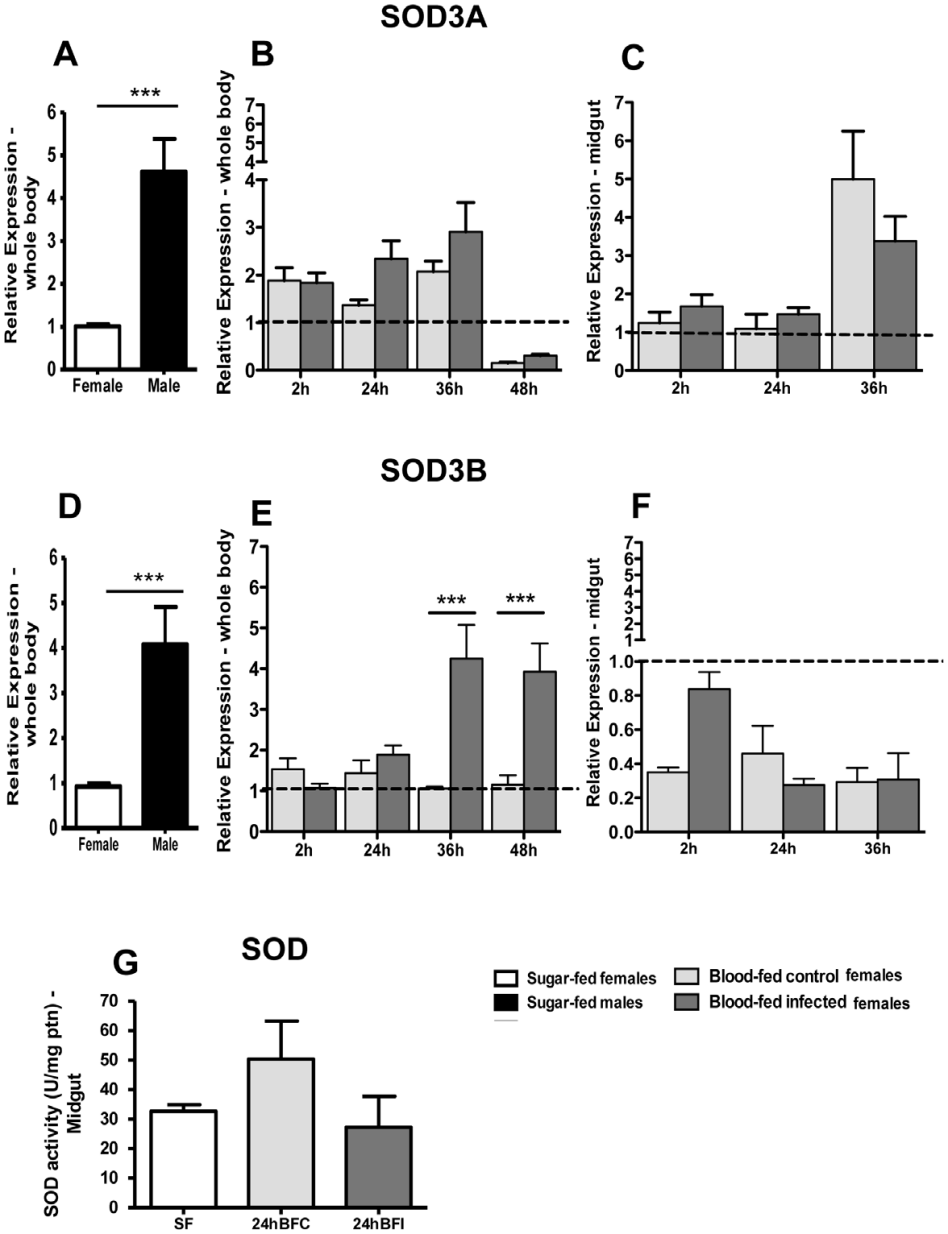
Expression levels and activity of SOD3A and SOD3B in *A. aquasalis* according to gender and feeding regimens. A and D: mRNA relative expression of SOD3A (A) and SOD3B (E) in whole body of sugar-fed males and females, B and D: mRNA relative expression of SOD3A (B) and SOD3B (E) in whole body of sugar-fed females (dotted line), and females after blood feeding and after *P. vivax* infection. C and F: mRNA relative expression of SOD3A (C) and SOD3B (F) in midgut of sugar-fed females (dotted line), and females after blood feeding and after *P. vivax* infection. G: SOD activity in sugar-fed females, 24 h after blood feeding (BFC) and after *P. vivax* infection (BFI) reported as units per minute per micrograms of protein (U/mg ptn). Data are reported as the mean ± SEM. * p<0.05, ** 0.03>p>0.01, *** p<0.01. In figures A and D the data was analyzed by t-student or the Wilcoxon tests, in figures B,C, E and F by ANOVA test with multiple comparisons of Tukey or Games-Howell or Kruskal-Wallis test with multiple comparisons of Dunn's, and in figure G by ANOVA test with Dunnett's Multiple Comparison Test.

The expression of the two SODs was much higher in sugar fed male than female mosquitoes (Figure 4A and 4D). SOD3A expression was upregulated in the whole body of *P. vivax*-infected insects 24 and 36 h after blood feeding but this difference was only significant at 36 h (Figure 4B). The expression pattern of SOD3B was quite different from SOD3A: while expression stayed at basal levels in the whole body of blood fed mosquitoes in all times investigated, levels increased dramatically at 36 h after the infectious meal, staying elevated until 48 h (Figure 4E). In the mosquito midgut, SOD3A was not modulated 24 h after blood feeding or infection, but was upregulated 36 hours after ingestion of blood and had a small decrease 36 hours after *P. vivax* infection (Figure 4C). SOD3B had low expression in the mosquito midgut after feeding and infection compared to sugar-fed control, with a peak of transcription 2 hours after infection (Figure 4F). SOD activity in the midgut of *A. aquasalis* decreased 24 h after infection (Figure 4G) compared to control mosquitoes, although this difference was not significant.

### Catalase silencing enhances *A. aquasalis* susceptibility to *P. vivax* infection

**Figure 5:**
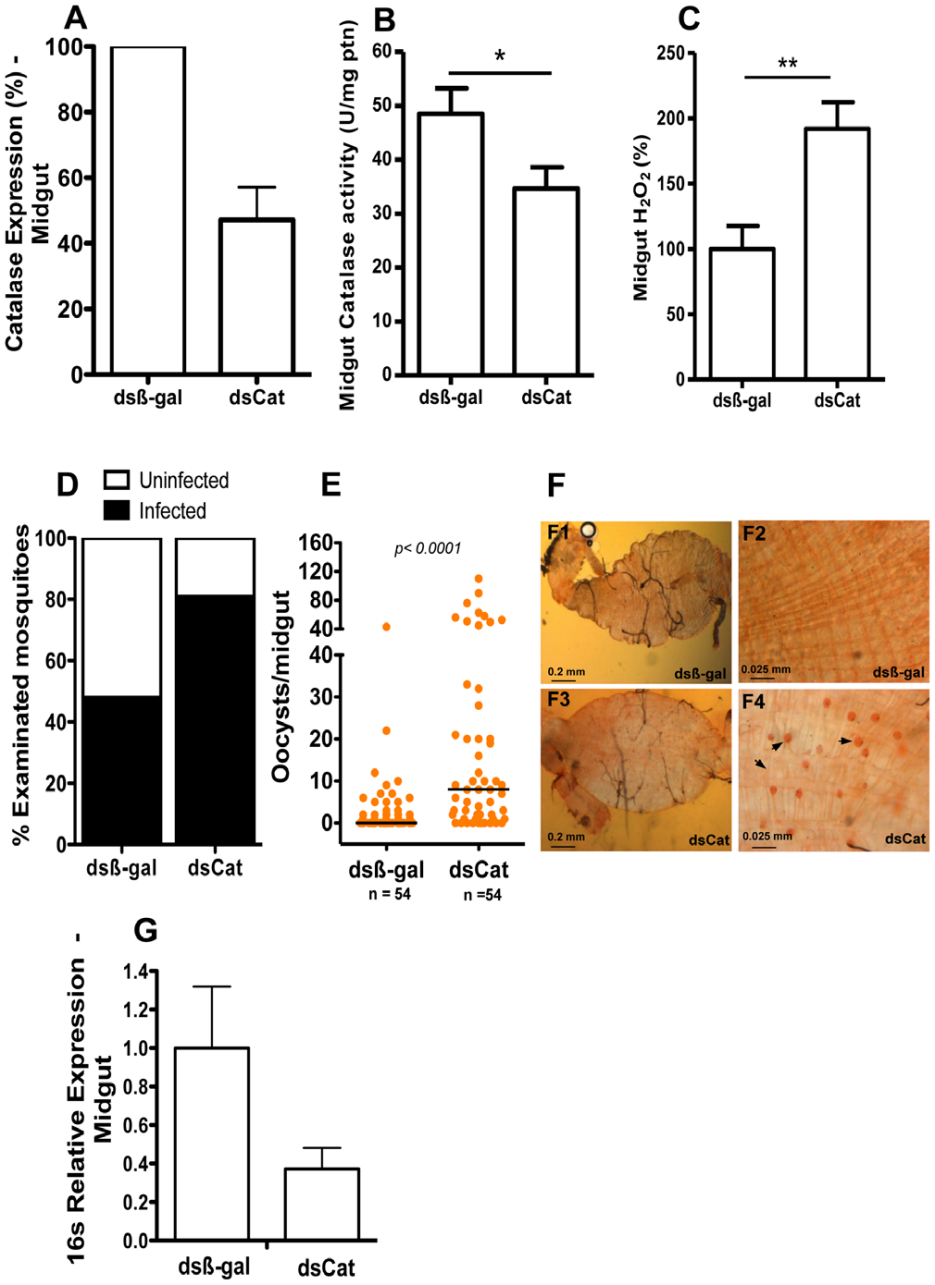
Molecular analysis of catalase silencing. A and B – Effect of dsCatalase (dsCat) injections on catalase mRNA expression (A) and activity (B) in the mosquito midgut. C – H_2_O_2_ production in the midgut of insects silenced for catalase and β-gal (control) genes. D – Prevalence of *P. vivax* infection in insects after β-gal and catalase dsRNA injections. E and F – Oocysts numbers in the midguts of dsβ-gal and dsCat injected mosquitoes 3–5 days after *Plasmodium* infection. The arrows show *P. vivax* oocysts in the *A. aquasalis* midgut. G: Bacterial load based on 16 s gene in the gut of mosquitoes silenced with dsCat and dsβ-gal. In figure A, data were analyzed by the ANOVA test with multiple comparisons of Tukey or Games-Howell or Kruskal-Wallis test with multiple comparisons of Dunn's; in figures B and C by unpaired t-test; in the figure E by the Mann-Whitney test.

To evaluate the effect of catalase knockdown on *A. aquasalis* infection by *P. vivax*, expression was reduced systemically by dsRNA-mediated silencing. Approximately 50% reduction of mRNA levels in insect midguts was achieved 2–3 days after dsRNA inoculation (Figure 5A). To assess the biological effects of RNAi-mediated gene silencing, we measured catalase activity in the midgut epithelium. The result shown in Figure 5B demonstrated a significant decrease in catalase activity 24 h after gene silencing suggesting a decreased H_2_O_2_ removing capacity in the midgut. In agreement, H_2_O_2_ release from the epithelia of sugar-fed mosquitoes injected with dsCat was significantly higher compared to dsβ-gal injected ones (Figure 5C), corroborating the efficiency and specificity of catalase silencing in the midgut of *A. aquasalis*. Considering several previous reports demonstrating the role of antioxidant enzymes, including catalase, in the response of *A. gambiae* to the murine malaria parasite *P. berghei* [19], [20], we investigated the effect of catalase silencing in *A. aquasalis* response to *P.vivax*. Surprisingly, catalase knockdown increased the percentage of infected insects (Figure 5D) as well as the number of oocysts in insect midguts (Figures 5E and 5F). Catalase inhibition by Aminotriazole also increased *A. aquasalis* susceptibility to *P. vivax* (Figure S3). To evaluate the possible reasons for this phenomenum, we investigated bacteria proliferation in the midgut of dsCat insects compared to controls. We observed a decrease bacterial load after catalase knock-down (Figure 5G).

## Discussion

Although *A. aquasalis* is an important malaria vector in Brazil [33] and *P. vivax* is the prevalent malaria parasite in the Americas, being responsible for half of the malaria cases outside the African continent, there is a lack of information on this parasite-vector pair [34]. This is mostly due to the absence of an efficient parasite cultivation system and the wrong assumption that this parasite does not cause severe and lethal malaria [35]–[37]. Some studies on Old World anopheline species and *P. falciparum* or non-human malaria parasites have shown that the mosquito immune system is responsible for healing infections and even conferring *Plasmodium* refractoriness [19], [38]–[40]. We recently reported the involvement of few immune genes in early infection of *A. aquasalis* by *P. vivax*, leading us to focus on specific immune targets [22]. We thus implicated the JAK-STAT pathway in this vector-parasite interaction [2].

Here we describe the role of ROS in the *A. aquasalis* response to *P. vivax*. ROS are important effector molecules that participate in the immune responses of organisms as diverse as mammals and insects against various pathogens [6], including mosquito response to *Plasmodium* [20]. SOD and catalase act together to detoxify superoxide and hydrogen peroxide. These two oxygen reactive species contribute to the formation of hydroxyl radical - the most oxidizing oxygen free radical - in the presence of iron [41], [42]. Here, the expression of three detoxification enzymes, catalase, SOD3A, and SOD3B, was characterized in relation to *A. aquasalis* gender and feeding regimens. *A. aquasalis* catalase is orthologous to other mosquito catalase genes, while clearly differing from the so-called immune-related catalase of *D. melanogaster* [32]. AqCAT mRNA expression increased in the mosquito whole body at 24 h after the blood meal, although at this point no significant difference was observed between infected and non-infected insects. A significant increase in catalase expression was observed at 36 h after infection. In the mosquito midgut catalase expression was upregulated by the blood meal, but no differences were found related to *P. vivax* infection. An increase in catalase mRNA expression was also observed in *A. gambiae* midgut tissues 24 h after ingestion of blood, although this expression decreased in midguts of infected mosquitoes [20]. In *A. aquasalis* infected with *P. vivax*, catalase activity significantly decreased at 24 h. Molina-Cruz and collaborators [20] also observed a decrease in *A. gambiae* catalase activity in the midgut 24 h after *P. berghei* infection. Differently from *P. berghei* infected *A. gambiae*, catalase mRNA of *A. aquasalis* was not downregulated in the midgut or in the moquito whole body 24 h after *P. vivax* infection, althought the activity was decreased at that time as obseerved in *A. gambiae* [20]. This indicates that in *A. aquasalis*,the manipulation of the detoxification system by the parasite only happens post-transcriptionally.

We characterized two *A. aquasalis* SODs, one related to SOD3A and the other to SOD3B of *A. gambiae*, showing quite different expression patterns. In whole mosquitoes, 24 h after infection with *P. vivax* only AqSOD3A expression increased significantly in relation to blood fed insects. And, although expression of both SOD genes increased in infected insects at 36 h, this difference was only significant for AqSOD3B. At 48 h after infection the difference was also impressive, with AqSOD3A expression falling to control blood fed levels, while SOD3B maintained the significantly high levels reached at 24 h of infection. In the mosquito midgut, the expression of SOD3A at 24 h had similar levels in infected and control insects, increased 36 h after blood-feeding, and again was not altered by infection. The expression of SOD3B was very low in the midgut, not reaching the level of sugar-fed controls. AqSOD activity decreased in the midgut 24 h after infection compared to blood fed control mosquitoes, although the difference was not significant. This may be due to the cumulative measurement of other SODs as SOD1 and SOD 2. The levels of mRNA for the two antioxidant enzymes catalase and SOD3B increased in the whole insect upon infection, which might be understood as an attempt of the mosquito to return free radicals to normal levels in their body, possibly counteracting increasing production by the activated immune system. However, when enzyme activity was measured in the midgut, both catalase and SOD showed reduced activity after infection. In our attempts to silence catalase we obtained silencing of midgut. Catalase silencing had a significant effect on catalase activity and in hydrogen peroxide levels in insect guts. Surprisingly, catalase knockdown exacerbated the infection of *A. aquasalis* by *P. vivax*. The opposite was reported for *P. berghei* parasites and *A. gambiae* [20], suggesting important functional differences regarding *Plasmodium* immunity in the *A. aquasalis* – *P. vivax* pair. Possible explanations are that the levels of ROS generated in the midgut of *A. aquasalis* are relatively low and do not compromise *P. vivax* survival or that *P. vivax* may have a much higher ability to detoxify ROS than *P. berghei*. We observed a decrease of natural microbiota in the mosquito midgut after catalase silencing. It is also possible that catalase knockdown led to the increase of ROS causing a decrease of competitive bacteria, thus allowing better *P. vivax* development inside the *A. aquasalis* mosquitoes. A similar situation was seen in *Salmonella typhimurium* infection of the mammalian gut [43]. These bacteria use the reactive oxygen species generated during inflammation to react with endogenous compounds generating a growth advantage for *S. typhimurium* over the competing microbiota in the lumen of the inflamed gut. It was also shown that oxidative stress generated in response to infection by the parasite *T. cruzi* contributes to maintenance of high parasite burdens in human macrophages [44].

In conclusion, upregulation of detoxifying enzyme genes in the mosquito whole body at 36 h after infection, when the parasite is fixing in the basal lamina and thus exposed to the haemolymph, may be due to expression in fat body and hemocytes.

On the other hand, the decrease of *A. aquasalis* catalase activity 24 hours after infection can be a consequence of the manipulation by the parasite to increase ROS, decrease the competitive microbiota and inhibit some immune pathways in order to improve its development inside the vector.

## Conclusions

The interactions between *Anopheles* insects and *Plasmodium* determine the ability of these mosquitoes to transmit malaria. In previous work, analyses of some immune genes showed that the presence of *P. vivax* in *A. aquasalis* haemolymph, rather than in the midgut or during passage through the midgut epithelium, appeared to correlate with the induction of an anti-microbial immune response [2], [22]. Here we showed that *P. vivax* initial infection decreased catalase activity and that catalase silencing increased the *P.vivax* parasites in the *A. aquasalis* midgut in a manner that apparently was not coherent with the model proposed of ROS-induced parasite killing. We propose here that *P. vivax* in the midgut probably manipulates the free radicals detoxification system of *A. aquasalis* and, as a consequence, control some competitive bacteria allowing better parasite development.

## Supporting Information

Figure S1 **Sequence of** ***A. aquasalis*** **catalase.** Numbers on the left represent nucleotide sequence length and on the right amino acid sequence length; asterisk indicates the stop codon; aminoacids in bold indicate the heme binding pocket; underlined aminoacids represent the tetramer interface. AqCAT sequence was deposited in GenBank with accession number HQ659100.
(TIF)

Figure S2 **Sequence of SOD3A (A) and SOD3B (B) cDNAs.** Numbers on the left represent nucleotide sequence length and on the right indicate amino acid sequence length; asterisk indicates the stop codon; underlined deduced aminoacids show the P-class dimer interface and in italics the E-class dimer interface; aminoacids in bold indicate aminoacids represent the active sites. AqSOD3A and SOD3B sequences were deposited in GenBank with accession numbers HQ659101 and HQ659102, respectively.
(TIF)

Figure S3 **Effect of** ***A. aquasalis*** **catalase inhibition by Aminotriazole on** ***P. vivax*** **oocysts development.** The data were analyzed by the Mann-Whitney test.
(TIF)
