# Supplementary figures and images for: The Role of Reactive Oxygen Species in Anopheles aquasalis Response to Plasmodium vivax Infection
Source: PLoS One. 2013 Feb 18;8(2):e57014. doi: 10.1371/journal.pone.0057014 (PMC3575503; doi:10.1371/journal.pone.0057014)

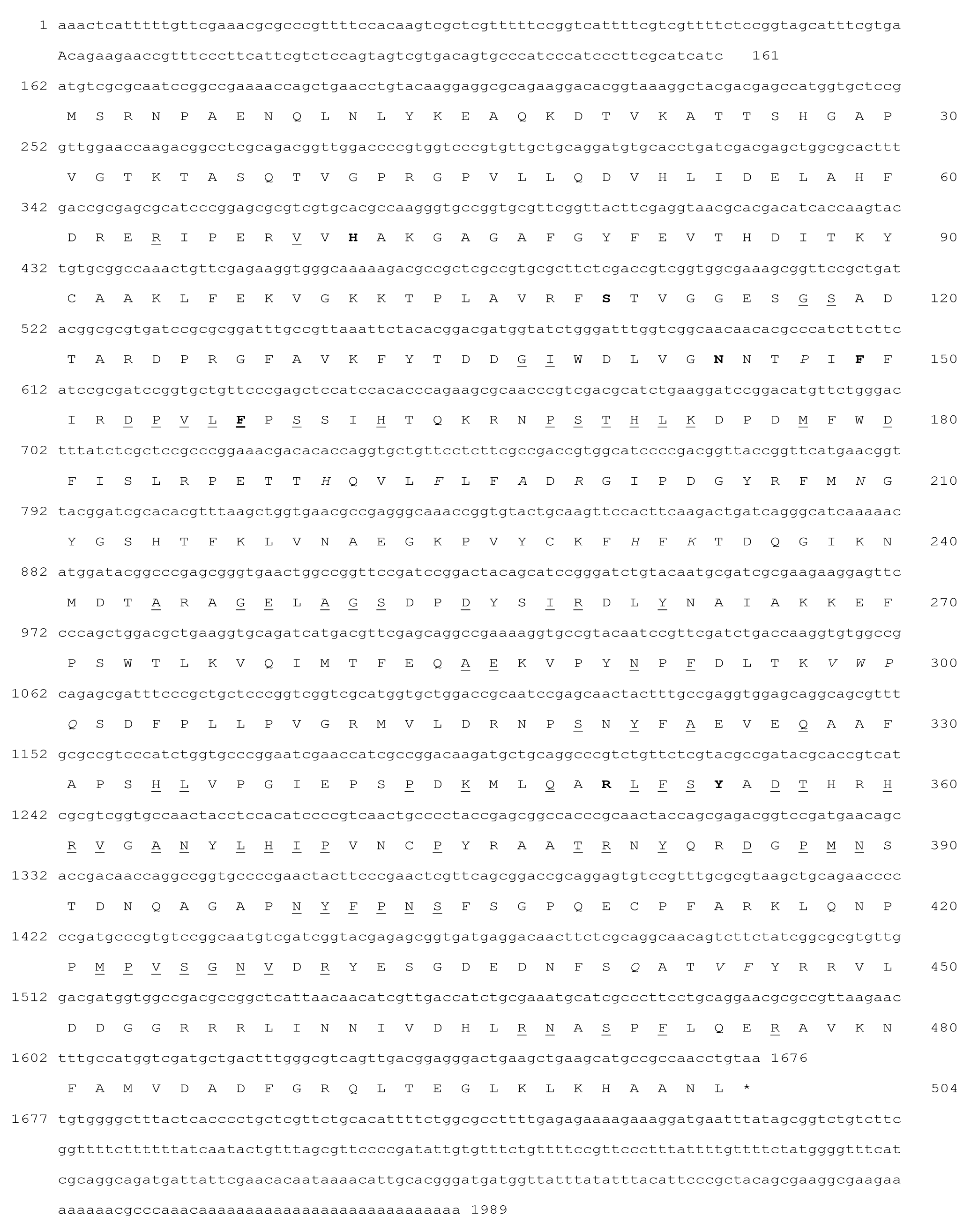

Supplement: Figure S1 — Sequence of A. aquasalis catalase. Numbers on the left represent nucleotide sequence length and on the right amino acid sequence length; asterisk indicates the stop codon; aminoacids in bold indicate the heme binding pocket; underlined aminoacids represent the tetramer interface. AqCAT sequence was deposited in GenBank with accession number HQ659100. (TIF) [file pone.0057014.s001.tif]

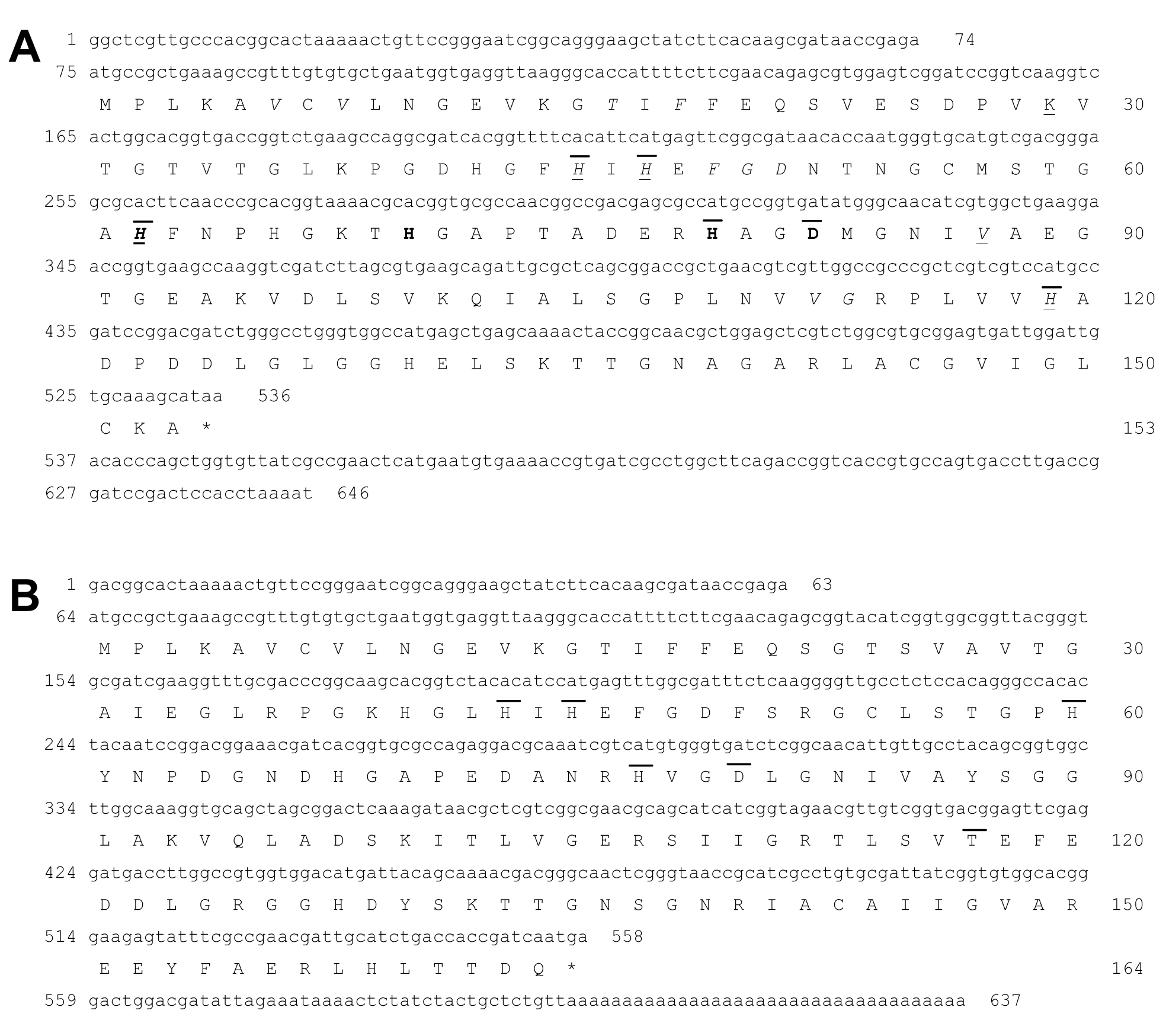

Supplement: Figure S2 — Sequence of SOD3A (A) and SOD3B (B) cDNAs. Numbers on the left represent nucleotide sequence length and on the right indicate amino acid sequence length; asterisk indicates the stop codon; underlined deduced aminoacids show the P-class dimer interface and in italics the E-class dimer interface; aminoacids in bold indicate aminoacids represent the active sites. AqSOD3A and SOD3B sequences were deposited in GenBank with accession numbers HQ659101 and HQ659102, respectively. (TIF) [file pone.0057014.s002.tif]

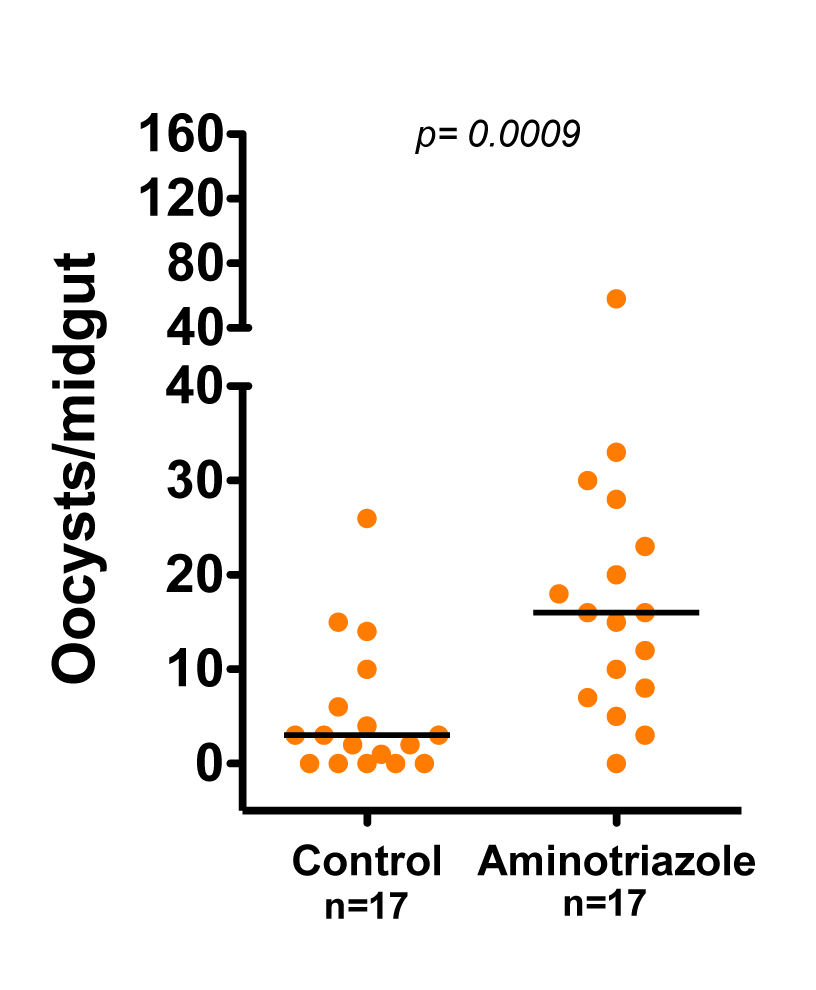

Supplement: Figure S3 — Effect of A. aquasalis catalase inhibition by Aminotriazole on P. vivax oocysts development. The data were analyzed by the Mann-Whitney test. (TIF) [file pone.0057014.s003.tif]
